# The First 40 Years of Molecular Ophthalmology Part Two: Diseases of the Primary Cilium and Heritable Glaucoma: The 2026 Friedenwald Lecture

**DOI:** 10.1167/iovs.67.12.81

**Published:** 2026-10-30

**Authors:** Val C. Sheffield

**Affiliations:** Departments of Pediatrics and Ophthalmology and Visual Sciences, University of Iowa, Iowa City, Iowa, United States

**Keywords:** animal models, molecular genetics, glaucoma, ciliopathies, retinopathies

##  

The identification of genes causing hereditary blindness has been ongoing for 40 years. Early studies to identify such genes began with using polymorphic genetic markers to map the location of the disease-causing gene. Our laboratory participated in the identification of human polymorphic markers and the creation of genetic maps, which were instrumental in disease mapping studies.^1^^,^^2^ Also instrumental for disease mapping studies was the identification and enrollment of multiplex families segregating specific diseases. As recognized by Sir Archibald Garrod in the early 1900s, rare autosomal-recessive disorders often result from consanguineous unions.^3^ To quote Garrod: “The question of the liability of children of consanguineous marriages to exhibit certain abnormalities or to develop certain disease has been much discussed…. There is no reason to suppose that mere consanguinity of parents can originate such a condition as alkaptonuria in their offspring, and we must rather seek an explanation in some peculiarity of the parents, which may remain latent for generations, but which has the best chance of asserting itself in the offspring of the union of two members of a family in which it is transmitted.^4^” We had the privilege and opportunity of collaborating with Drs. Rivka Carmi and Khalil Elbedour of the Ben Gurion University of the Negev in Be'er Sheva, Israel. They worked with Bedouin populations that have a high incidence of consanguinity and identified three large consanguineous families segregating Bardet-Biedl syndrome (BBS).^5^^–^^8^

BBS is a syndromic, heterogeneous, autosomal-recessive disorder comprising a constellation of clinical findings, including retinal degeneration, obesity, polydactyly (Fig. 1), hypogonadism, renal abnormalities, and learning disabilities.^9^^,^^10^ BBS displays a high level of genetic heterogeneity.^9^^–^^11^ Since the identification of linkage to the first BBS locus by our laboratory in 1993 using a large Bedouin kindred,^6^ great progress has been made in identifying 25 genes (to date) that can independently cause BBS when mutated.^12^^–^^31^

**Figure 1.**
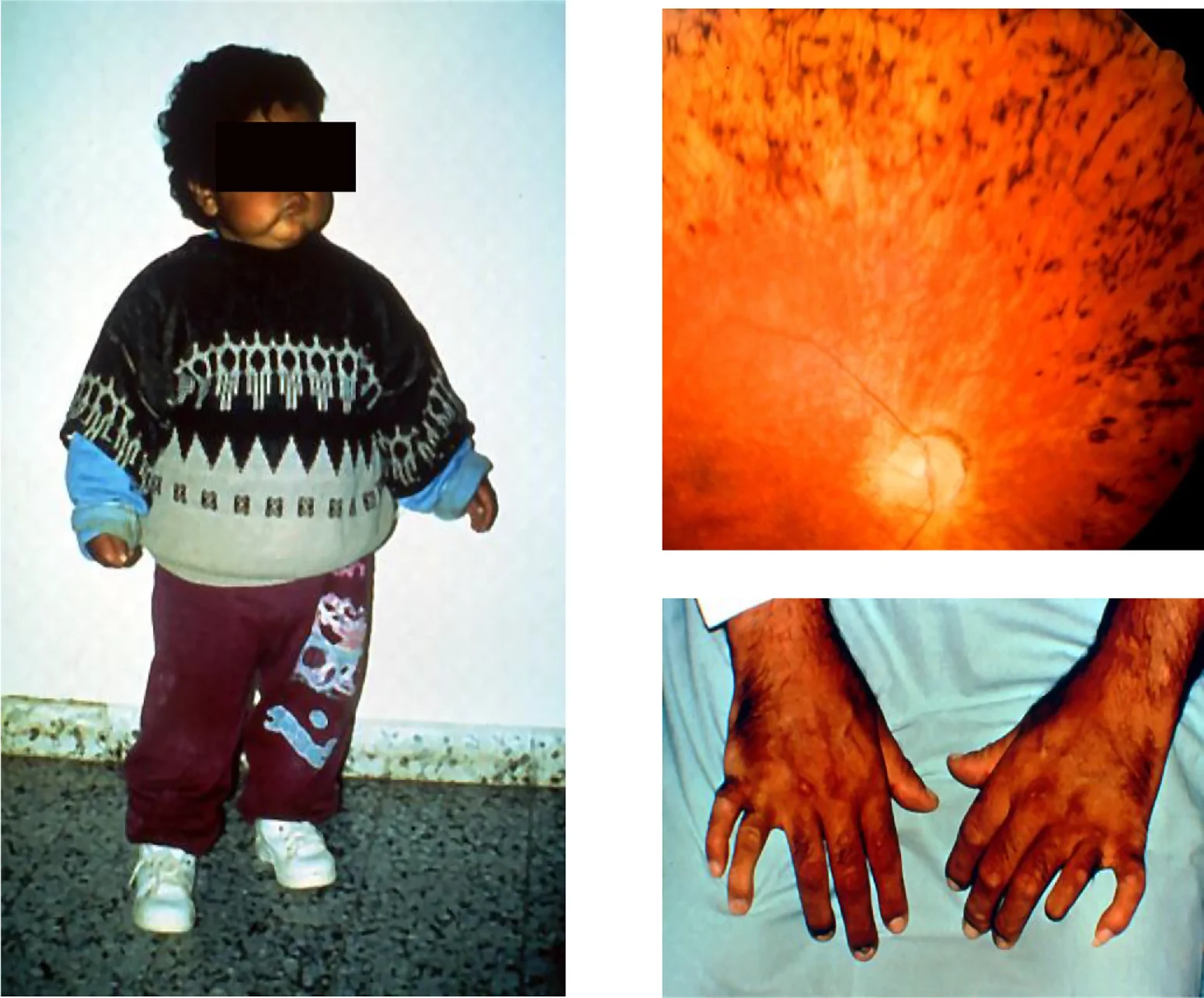
An obese BBS patient (*top left*), retina with bone spicule pigmentation (*top right*), and polydactyly (*bottom right*).

After several BBS genes had been identified, the function of the proteins coded for by these genes was not clear. We hypothesized that the proteins form a complex or complexes and used tandem affinity purification to look for complexes. Eight of these genes code for proteins that form a stable complex referred to as the BBSome consisting of BBS1, BBS2, BBS4, BBS5, BBS7, BBS8, BBS9, and BBS18.^32^^–^^34^ Three other BBS proteins (BBS6, BBS10 and BBS12) have homology to chaperonins and form a chaperone complex that plays a role in the assembly of the BBSome complex.^35^ When these proteins are absent or mutated, the BBSome is not formed normally.

To investigate the function of these multimeric protein complexes, we created several mouse models including models for BBS1, BBS2, BBS4, BBS6, and BBS7.^36^^–^^41^ These models provided the insight that BBS proteins are involved in cilia formation and function. These mouse models all have similar features, including spermatozoa lacking flagella, airway cilia that are present but abnormal, and photoreceptors that die due to having a disorganized outer segment, which is a specialized primary cilium. The current model for BBSome function is that the BBSome serves as an adapter for retrograde transport to export proteins from the cilia.

Another BBS protein, BBS3 (also known as ARL6), is an Arf-like GTPase that plays a role in recruitment of the BBSome to membranes in its GTP-bound state.^19^^,^^41^ The absence of functional BBS3 results in a failure of the BBSome to properly localize to cilia.^41^ Another BBS protein, BBS17 (LZTFL1), negatively regulates BBSome ciliary trafficking, that is, LZTFL1 prevents the BBSome from entering cilia.^27^ Mutations in the genes involved in the formation and function of the BBSome cause the vast majority of BBS cases. Furthermore, nearly all BBS patients with mutations in these genes develop retinal degeneration at a relatively young age. The loss of vision is the aspect of BBS that has the greatest impact on the quality of life of BBS patients and creates a large economic burden to patients and society.

To gain more information about the BBSome and its role in photoreceptors, we created a BBS8 mouse model in which BBS8 expression is suppressed by a gene trap.^42^ The gene trap can be removed by using tamoxifen to induce the expression of a recombinase, which results in the formation of the BBSome. This can be done at various times. Before removal of the gene trap, the outer segments are abnormal with a highly disorganized disc morphology. Once the gene trap is removed, BBS8 is expressed and the BBSome is restored. Restoration of the BBSome results in a normal disc morphology and rescue of the remaining photoreceptors (Fig. 2).

**Figure 2.**
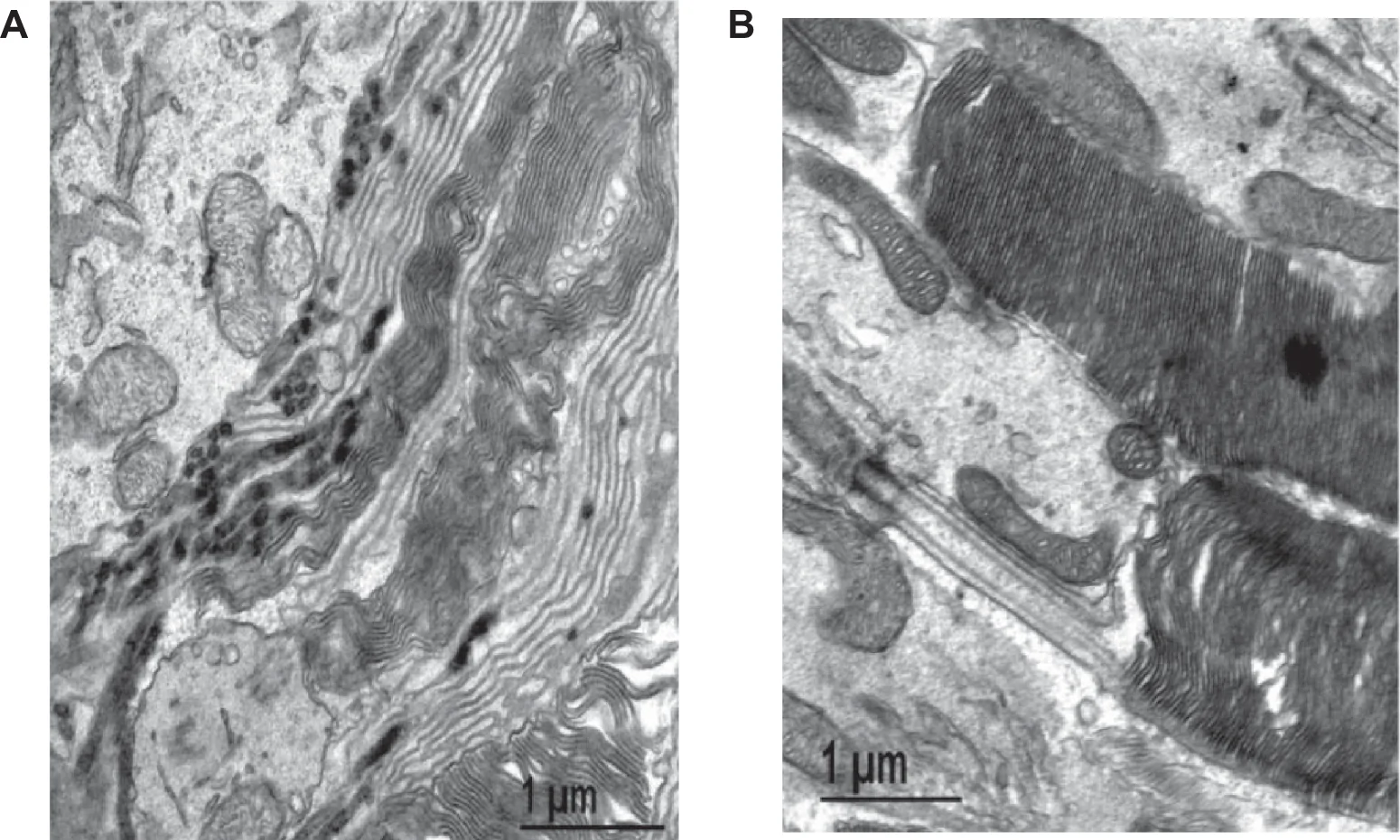
(**A**) Disorganized photoreceptor disc morphology when *Bbs8* expression is suppressed by the gene trap. (**B**) Normal disc morphology after initiation of *Bbs8* expression upon removal of the gene trap in 2-month-old mice.

In addition to BBS, our laboratory has worked on glaucoma. Glaucoma is an optic neuropathy that leads to ganglion cell death. It is a leading cause of irreversible blindness affecting more than 1% of the world population.^43^ There are various types of glaucoma with POAG being the most prevalent type. An elevated IOP is a major risk factor. We had the opportunity to use a large Mid-Western family that has an autosomal-dominant juvenile-onset form of POAG. We used polymorphic genetic markers to map the disorder to chromosome 1 in 1993.^44^ It took nearly 4 years to use positional cloning methods to identify the disease gene.^45^ The gene involved is called *MYOC*, which codes for the myocilin protein, which is expressed in the trabecular meshwork. The mutations causing POAG are found at the carboxy-terminus coded for by exon 3 of the three-exon gene (Fig. 3). *MYOC* is involved in 3% to 5% of adult-onset POAG, which is the most of any known genetic form of glaucoma.^46^

**Figure 3.**
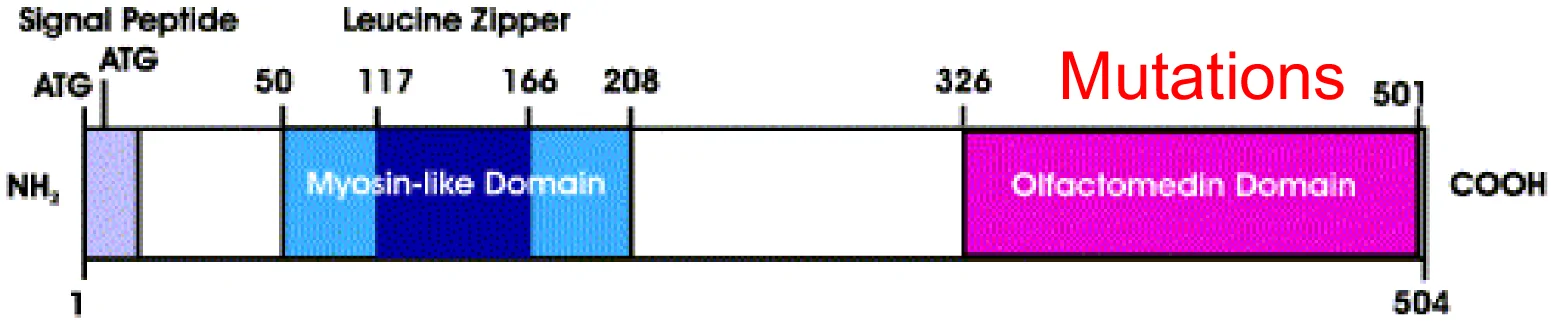
The myocilin protein coded for by the *MYOC* gene. The mutations causing *MYOC*-associated primary open-angle glaucoma are found at the carboxy-terminus.

We and others made several attempts to create a mouse model. The overexpression of mouse *Myoc* results in no phenotype. Knock out of the gene also causes no phenotype. Knocking in a mutation into the mouse gene also results in no phenotype. We were finally able to create a mouse model by humanizing the mouse, that is, by expressing a mutant human *MYOC* gene in the mouse.^47^ This led to an elevated IOP beginning after 1 month of age. In addition, the expression of the mutant human myocilin results in a loss of retinal ganglion cells and function, as well as a loss of optic nerve axons. The mechanism by which mutant human myocilin causes glaucoma is that the mutant protein misfolds and accumulates intracellularly resulting in endoplasmic reticular stress. Endoplasmic reticular stress leads to trabecular meshwork disfunction and an elevated IOP, resulting in ganglion cell death.

Using the mouse model, we developed a treatment using the CRISPR-Cas9 system.^48^ We reasoned that a guide RNA targeting a position early in the gene would result in nonhomologous end joining resulting in insertions or deletions leading to an early stop codon, preventing a protein from being formed. We reasoned that it would not matter if both the mutant and the normal copy were knocked out, because having no myocilin does not create a phenotype. We performed intravitreal injections of MYOC CRISPR-Cas9 in the mouse model. This treatment can be performed in young mice before they develop elevated IOPs, preventing IOP elevation (Fig. 4A). Treatment can also be performed in older mice that have elevated IOPs, resulting in a lowering of the IOP (Fig. 4B). We have also shown that we can use CRISPR treatment to remove myocilin in human donor eyes, indicating that this treatment should work in humans.^48^

**Figure 4.**
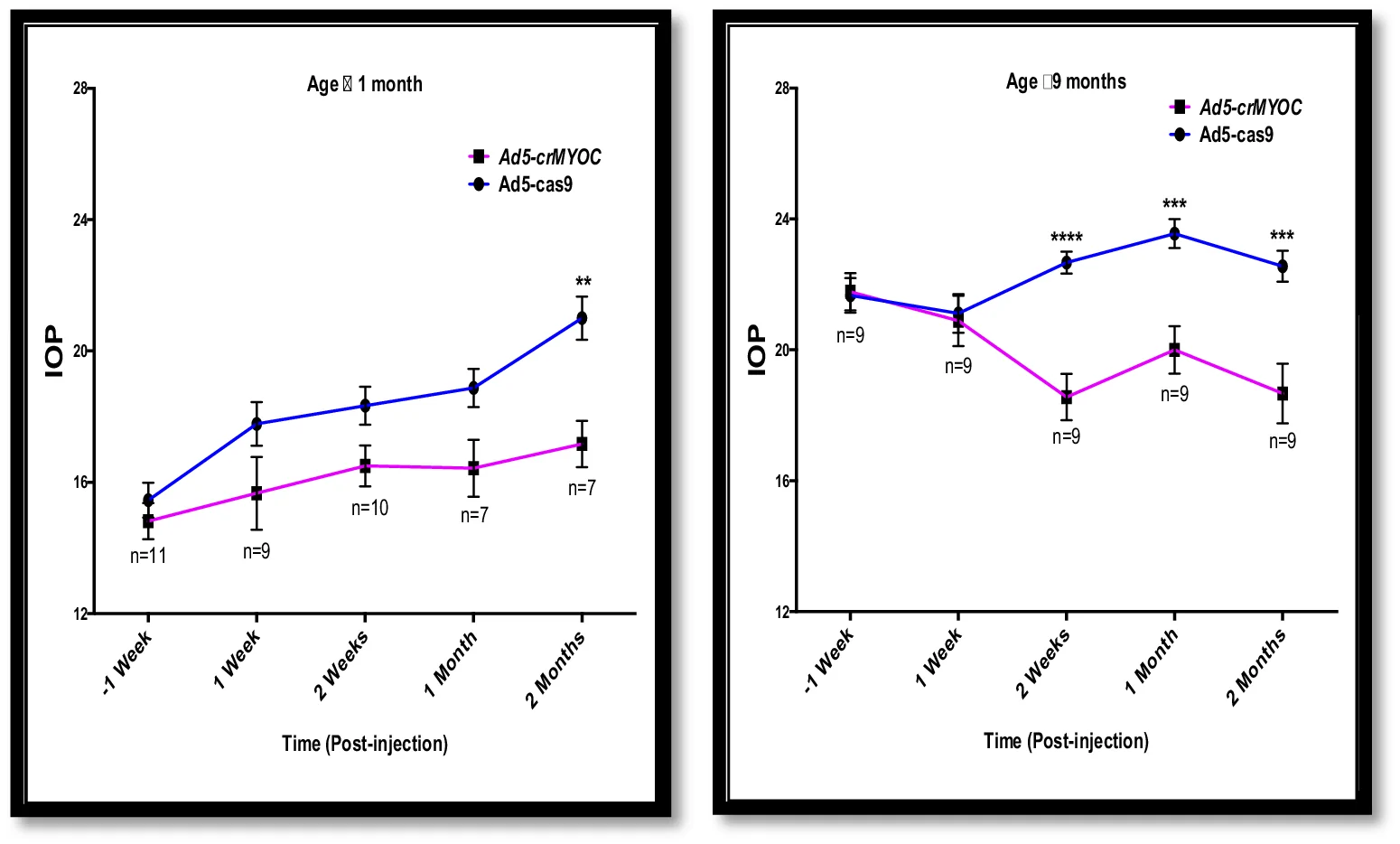
(*Left*) Results of treating ∼1-month-old mice with MYOC CRISPR-Cas9 preventing an elevation of the IOP. (*Right*) Results of treating ∼9-month-old mice with MYOC CRISPR-Cas9 resulting in a reduction of the IOP.

There are numerous oxidative stress–related disorders that have an abnormal redox state including diseases related to the eye such as diabetes. We made a serendipitous discovery that we could use electromagnetic fields (EMFs) to treat diabetes.^49^ These are specific static fields that include an electric field with an orthogonal magnetic field. We initially demonstrated using BBS mice that EMF normalizes blood sugar. We also demonstrated that this works in more traditional mouse models of diabetes, including high-fat diet-fed mice, which become obese and develop diabetes, and in db/db mice, which lack the leptin receptor. The db/db mice become very obese and develop diabetes. This works by EMF exerting a force on superoxide radicals, which are paramagnetic. Superoxide is converted to hydrogen peroxide catalyzed by superoxide dismutase. The EMF increases the production of hydrogen peroxide from superoxide. In the untreated state, diabetic mice have higher than normal levels of reactive oxygen species, resulting in a more oxidized state and decreased insulin sensitivity. Treatment with EMF results in a decrease in reactive oxygen species, a reduced redox state, and increased insulin sensitivity, resulting in normalization of the blood sugar (Fig. 5).

**Figure 5.**
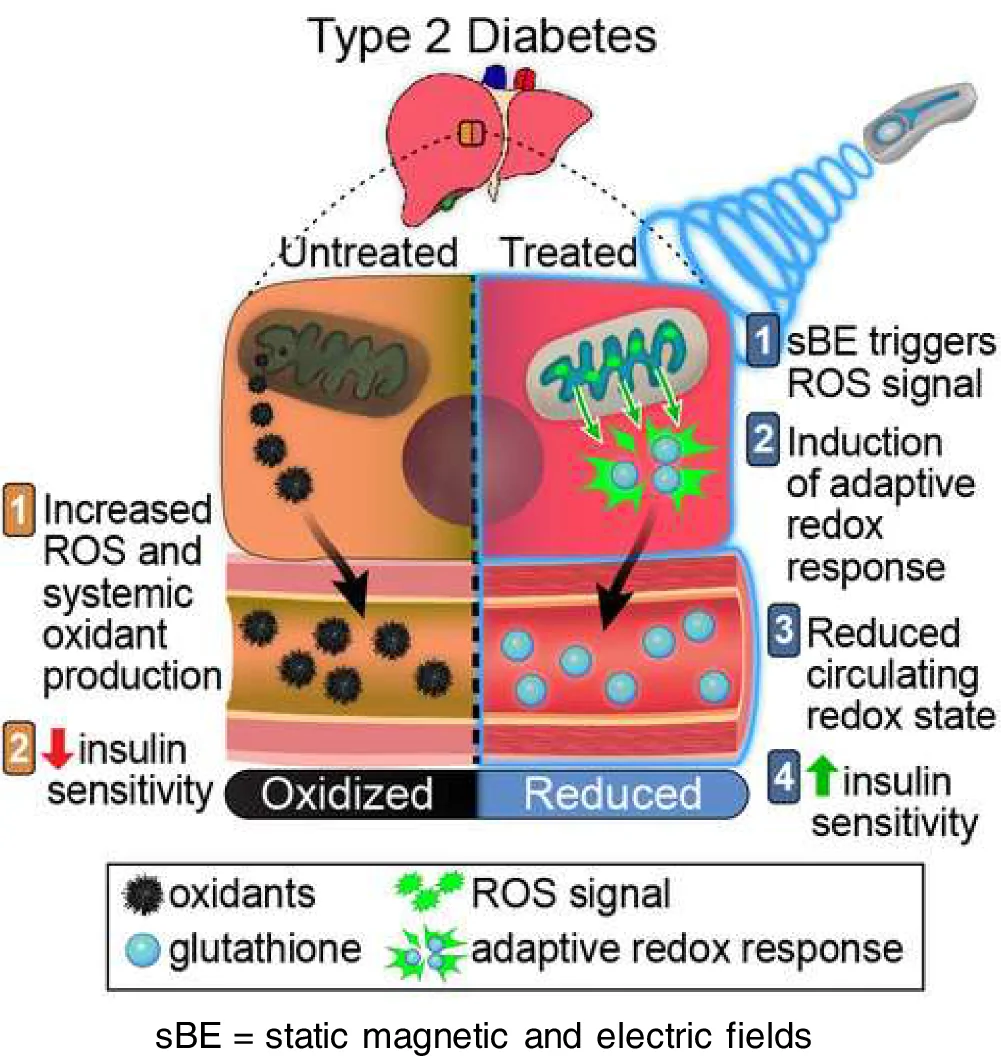
The schematic shows that static magnetic (**B**) and static electric (**E**) fields, jointly referred to as sBE, results in the oxidized state of diabetic mice becoming a more reduced state leading to increased insulin sensitivity. ROS, reactive oxygen species. Reprinted from Cell Metabolism 2020;32(4):561–574, with permission from Elsevier.

There are significant amounts of literature indicating that oxidative stress plays a role in eye diseases, including glaucoma, AMD, diabetic retinopathy, and other retinopathies.^50^^–^^56^ We believe that EMF can be used to treat glaucoma and retinopathies. Experiments are ongoing to demonstrate this.
